# Activator Protein-1 Transcriptional Activity Drives Soluble Micrograft-Mediated Cell Migration and Promotes the Matrix Remodeling Machinery

**DOI:** 10.1155/2019/6461580

**Published:** 2019-12-31

**Authors:** Martina Balli, Jonathan Sai-Hong Chui, Paraskevi Athanasouli, Willy Antoni Abreu de Oliveira, Youssef El Laithy, Maurilio Sampaolesi, Frederic Lluis

**Affiliations:** Department of Development and Regeneration, Stem Cell Institute, KU Leuven, B-3000 Leuven, Belgium

## Abstract

Impaired wound healing and tissue regeneration have severe consequences on the patient's quality of life. Micrograft therapies are emerging as promising and affordable alternatives to improve skin regeneration by enhancing the endogenous wound repair processes. However, the molecular mechanisms underpinning the beneficial effects of the micrograft treatments remain largely unknown. In this study, we identified the active protein-1 (AP-1) member Fos-related antigen-1 (Fra-1) to play a central role in the extracellular signal-regulated kinase- (ERK-) mediated enhanced cell migratory capacity of soluble micrograft-treated mouse adult fibroblasts and in the human keratinocyte cell model. Accordingly, we show that increased micrograft-dependent *in vitro* cell migration and matrix metalloprotease activity is abolished upon inhibition of AP-1. Furthermore, soluble micrograft treatment leads to increased expression and posttranslational phosphorylation of Fra-1 and c-Jun, resulting in the upregulation of wound healing-associated genes mainly involved in the regulation of cell migration. Collectively, our work provides insights into the molecular mechanisms behind the cell-free micrograft treatment, which might contribute to future advances in wound repair therapies.

## 1. Introduction

The skin is the largest organ of the human body providing protection, immune defense, and sensation. Traumatic injuries and impaired wound healing greatly affect the quality of life of patients and eventually contribute to mortality. Therefore, optimal tissue regeneration and proper wound management are required to maintain skin integrity.

The process of wound healing emerges as a result of a complex interplay between transcription factors that regulate essential transcriptional networks. Their activation is tightly regulated by signaling cascades that translate the input of intrinsic and extrinsic factors into a transcriptional cellular response to such cellular changes. During wound healing, sequential cellular processes are actively coordinated by waves of growth factors and cytokines [1–3]. Upon injury, several transcription factors are triggered by the damaged tissue. Among those, TGF-*β* signaling and the classical transcription factor NF-*κ*B have been described as regulators which elicit the immune response as the initial step of the wound healing process. This results in the immune cell and platelet-derived production of a plethora of growth factors including transforming growth factor-beta (TGF-*β*), epidermal growth factor (EGF), platelet-derived growth factor (PDGF), vascular endothelial growth factor (VEGF), and connective tissue growth factor (CTGF) at the site of injury [4–6]. These in turn establish key repair processes such as angiogenesis, matrix deposition, migration, proliferation, and fibroblast transdifferentiation through pivotal signaling cascades [4]. While TGF-*β* signaling is an essential regulator of wound contraction and matrix deposition by myofibroblasts, the MEK/ERK cascade has been reported to promote proliferation and migration in both keratinocytes and fibroblasts, the main cell types involved in wound healing [7–9]. Interestingly, the activator protein-1 (AP-1) family has been reported as a transcription factor downstream of the ERK cascade [10, 11]. Upon injury, AP-1 activity has been shown to be induced in an MAPK-dependent manner in fetal mouse skin [12]. This protein family consists of homo- and heterodimers formed mainly between proteins of the Fos and Jun families [13]. Upon phosphorylation, AP-1 is known to regulate genes involved in wound healing, such as growth factors and matrix metalloproteases (MMPs) [14]. Collectively, successful wound repair strongly depends on the spatiotemporal activity of several cellular signaling pathways.

The gold standard treatment to promote wound repair remains skin graft methods involving skin transplantations and have been widely used for the treatment of extensive wounds, such as burns, skin trauma, and cancer. As these lack blood vessels, additional blood support underneath the wound bed is required to guarantee the success of the skin graft. Moreover, this technique is associated with distress, pain, and hypertrophic scar formation [15]. Over the past years, great advances have been made in micrograft (MG) treatments, of which many have shown promising results in the clinical setting [16]. Compared to the conventional skin graft methods, the micrograft technique comprises of viable microtissue fragments that are directly applied to the site of injury. This autologous treatment has been reported to allow a 100-fold expansion of the micrograft transplant and offers a fast and reliable procedure for the patients with minimal surgical risks [17]. Recently, a novel MG technique has emerged as an innovative and affordable wound treatment whereby skin tissues are ground into a resulting solution of 80 *μ*m microtissue fragments containing tissue-residing progenitor cells and matrix components [18]. Moreover, a recent study has shown that skin injury treatment with the soluble MG fraction deprived of tissue-residing cells and highly enriched in growth factors (such as IGF-1 and bFGF) is sufficient to promote the endogenous regenerative capacity of the skin by accelerating wound closure, showing increased reepithelialization, and increasing granulation formation and increased CD31^+^ vessel formation [19].

Recently, the extracellular signal-regulated kinase (ERK) signaling pathway has been identified as one of the main drivers of the molecular mechanism behind this technique [19]. Upon MG treatment, both fibroblasts and keratinocytes displayed high levels of phosphorylated ERK (p-ERK) and exhibited upregulated transcription of MMPs. In accordance with this, the beneficial effects induced by the MG were reverted upon ERK inhibition. Nevertheless, a detailed molecular roadmap of the transcriptional network connecting ERK activation and downstream target expression and increased migratory capacity upon soluble MG treatment still remains largely unknown.

In this work, we dissect the molecular regulatory network behind the accelerated cell migration obtained by the MG treatment. To unveil key transcriptional regulators involved in the ameliorating effects mediated by this therapy, we integrated differentially expressed genes (DEGs) obtained upon a 5 h treatment with the noncellular micrograft solution into a gene regulatory network analysis (GSE123829) [19]. Interestingly, MG-dependent DEGs appeared to be likely regulated by important members of the AP-1 transcription factors, such as Fra-1. Accordingly, MG-induced fibroblast and keratinocyte migration as well as enzymatic activity of MMPs are significantly impaired upon AP-1 inhibition. Finally, our findings provide evidence that AP-1 connects higher ERK activity with increased MMP activity, which in turn regulates cell migration in both fibroblasts and keratinocytes. Collectively, our study provides a deeper view into the molecular mechanism behind the MG treatment, revealing the significance of AP-1 and its vital role in current and future advances in wound healing therapies.

## 2. Methods

### 2.1. Cell Culture

Tail tips (0.5 cm) of the C57BL/6 mouse model under proper anesthesia (isoflurane) were used to extract primary fibroblasts. Tissues were digested with collagenase IV for 40 minutes at 37°C. Cells were then cultured in DMEM GlutaMax (Cat No. 61965-026, Gibco Life Science, Grand Island) supplemented with 10% of fetal bovine serum, 1% penicillin-streptomycin, 1% L-glutamine, 1% sodium pyruvate, and 1% nonessential amino acids. The immortalized keratinocyte (HaCaT) cell line was used as a human keratinocyte cell model and was grown in Dulbecco's modified Eagles' medium (DMEM) supplemented equally to the previous media. Cells were cultured at 37°C in a humidified incubator with 5% of CO_2_.

### 2.2. Preparation of Micrografts

Micrografts were generated from murine skin biopsies (1 cm^2^) employing the Rigenera*®* technique. Tissues were minced using the Rigeneracons*®* tissue disruptor [20]. Resulting micrografts were subsequently filtered through 70 *μ*m filter membranes to obtain a protein concentration of 5 *μ*g/*μ*L, followed by a centrifugation step (10 min at 3000 rpm) to deplete the solution of residing cells and cellular debris. The extracted soluble micrograft was then stored at −80°C for later use. Subsequently, the soluble MGs were then diluted 1 : 10 in growth media and applied on fibroblast and keratinocytes cells. Signaling pathways and transcription factor AP-1 were altered by inhibitory drugs such as MEK inhibitor PD0325901 (1 *μ*M; Cat No. PZ016, Sigma) and c-fos/AP-1 inhibitor T-5224 (60 *μ*M; Cat No. 530141-72-1, MedChemExpress (MCE)), respectively.

### 2.3. Cell Scratch Assay

Fibroblasts and keratinocytes were plated at a density of 3500 cell/cm^2^ and 10000 cell/cm^2^, respectively. The scratch assay was performed on cell monolayers using a pipette tip, resembling an artificial *in vitro* wound [21]. Subsequently, dead cells were removed using sterile phosphate-buffered saline (PBS) and images were taken over a time course until full closure of the *in vitro* scratch was achieved. The comparison between images allowed quantification of the cell migration rate. MGs were added onto the cells for different treatment durations (1 h, 5 h, and 12 h).

### 2.4. Cell Cycle Assay and Cell Viability

Changes in the cell cycle were assessed using the 5-ethynyl-2′-deoxyuridine (EdU) assay (Thermo Fisher Scientific) [22]. MG-treated cells were incubated for 2 hours at 37°C with 10 *μ*M EdU (ethynyl-2′-deoxyuridine) according to the manufacturer's instructions (Cat. No 900584-50MG, Sigma-Aldrich). Samples were then examined by Flow Cytometry (BD Biosciences) and analyzed by FlowJo software. Viability of MG-treated cells was evaluated by flow cytometry (BD Biosciences) using the Fixable Viability Stain 660 (564405; BD Biosciences). Statistical analysis was performed using two-tailed unpaired *t*-tests and two-way analysis of variance (ANOVA). No significant differences were found (*n* = 3 biological replicates).

### 2.5. Immunohistochemistry, Immunofluorescence Staining, and Protein Analysis

Scratched cells and 60-70% confluent conditions were used for immunohistochemistry and immunofluorescence analysis. Cells were fixed with 4% PFA for 20 minutes at RT, permeabilized with 1% BSA, 0.2% Triton-X in PBS for 30 minutes at RT, washed repeatedly, and blocked with donkey serum 1 : 10 in PBS (blocking solution) for 40 minutes at RT. Cells were then incubated overnight at 4°C with the following primary antibodies: Rabbit mAb Phospho-Fra-1 (Ser265) (D22B1; CST5841T, Bioké-Cell Signaling) and Mouse mAb Fra-1 (D3; sc-376148, Santa Cruz Biotechnology) were used at a 1 : 200 dilution. Subsequently, cells were washed with PBS and incubated with secondary Alexa Fluor antibodies for 1 h at RT. Finally, cells were incubated in Hoechst 33342 at a 1 : 10000 dilution for nuclear counterstaining. Finally, cells were mounted using the DAKO™ mounting medium. Images were acquired using a Zeiss AxioImager Z1 Microscope and the AxioVision SE64 software. Images were analyzed using the ImageJ software (NIH).

Western blotting analyses were used to unveil the role of important proteins of the AP-1 complex in regulating the effects induced by the MG treatment. MG-treated cells were lysed in RIPA buffer containing 1 : 100 protease inhibitor and phosphatase inhibitor cocktails (Cat. No P8340-1 mL, P5726-1 mL, P0044-1 mL, respectively). Samples (30 *μ*g of proteins) were heat-denatured in sample-loading buffer (50 mM of Tris-HCl, pH 6.8, 100 mM DTT, 2% SDS, 0.1% bromophenol blue, 10% glycerol), resolved by SDS-PAGE and transferred to PVDF membranes (Cat. No 1620177, Bio-Rad). Membranes were then stained with Ponceau S dye (Sigma-Aldrich) and blocked with 5% BSA (bovine serum albumin) in TBS (Tris-buffered saline) supplemented with Tween 0.05% for 1 hour at RT. Membranes were then incubated overnight at 4°C with the following primary antibodies: mouse p-ERK (1 : 1000, sc-7383, Santa Cruz), rabbit ERK (1 : 1000, sc-94, Santa Cruz), pFra-1 (Phospho-Fra-1 (Ser265) (D22B1) Rabbit mAb; 1 : 1000, Cat. No CST5841T, Bioké-Cell Signaling), Fra-1 (Fra-1 Antibody (D-3) Mouse monoclonal, 1 : 1000, Cat. No sc-376148, Santa Cruz Biotechnology), pJun (Phospho-c-Jun (Ser73) (D47G9) XP® Rabbit mAb; 1 : 1000, Cat. No 3270 T, Bioké-Cell Signaling), JUN (c-Jun (60A8) Rabbit mAb; 1 : 1000, Cat. No 9165T, Bioké-Cell Signaling), and mouse Tubulin-alpha (1 : 1000, T5168, Sigma-Aldrich). The following day, membranes were incubated with secondary horseradish peroxidase HRP-conjugated antibodies (1 : 5000, Santa Cruz Biotechnology, CA, USA), in TBS 0.05% Tween and 2.5% nonfat dry milk. Membranes were stripped and reblotted for the total forms of Erk, Fra-1, and c-Jun. Analyses were performed using the Clarity Western ECL Substrate (Cat. No 1705060, Bio-Rad).

### 2.6. Matrix Metalloproteinase (MMP) Enzymatic Activity

MMP activity was detected using the MMP activity Assay Kit (Cat No. ab112146, Abcam), according to the manufacturer's instructions. Data is presented as RFU (relative fluorescence units). Following MG treatment, conditioned medium was collected after 24 hours. Signals were evaluated 30 minutes after initiation of the enzymatic reaction using a microplate reader with a filter set of Ex/Em = 485/535 and normalized to the substrate control.

### 2.7. Transwell Migration Assay

Cells were dissociated using 0.25% trypsin-EDTA, washed with PBS (1x), and pelleted. Multiple well plates with permeable polycarbonate membrane transwell inserts (Cat No. 3422, Thermo Fisher Scientific) were used for this experiment. Fibroblasts or keratinocytes (10^5^ cells) were plated on the upper compartment of the transwell in a total volume of 100 *μ*L of growth media. 600 *μ*L of 1 : 10 diluted MGs were applied to the lower chamber. 100,000 cells (fibroblast or keratinocytes) were submerged in 600 *μ*L of complete GlutaMax medium as a control. After incubation, the transwell inserts were removed, cleaned of stationary cells, and fixed with 70% ethanol. Afterwards, membranes were stained with 0.1% of crystal violet. Finally, membranes were detached from the inserts, deposited on slides, and mounted using DPX. Images were acquired using the AxioVision SE64 software on a Zeiss AxioImager Z1 Microscope. Images were analyzed using the ImageJ software (NIH).

### 2.8. RNA Extraction and mRNA Gene Expression

Total RNA was purified using the PureLink® RNA Mini Kit (Cat No. 12183018A, Life Technologies™) according to the manufacturer's instructions. cDNA was generated from 0.5 *μ*g of RNA and using the Superscript III Reverse Transcriptase First-Strand Synthesis SuperMix (Invitrogen). Quantitative real-time PCRs (qRT-PCR) were performed using the Platinum® SYBR® Green qRT-PCR SuperMix-UDG (Cat No. 11733038, Thermo Fisher Scientific) on a ViiA™ 7 Real-Time PCR System with 384-well plate (Cat No. 4453536, Applied Biosystems). Gene expression values were normalized to *GAPDH*, *Rpl13a*, and *β-actin* for mouse primary fibroblasts and *GAPDH*, *RPL13A*, and *β-actin* housekeeping genes for human keratinocytes. All primers used were purchased from IDT technologies, Leuven, Belgium, and are reported in [Supplementary-material supplementary-material-1].

### 2.9. RNA Sequencing and Bioinformatics Analyses

The data presented in this paper has been deposited in GEO Datasets under accession no. GSE134113. The integrity of the RNA was evaluated using the Bioanalyzer (Agilent 2100) combined with the Agilent RNA 6000 Nano Kit (Ca No. 5067-1511). Samples were processed by the Genomics Core Leuven (KU Leuven–UZ Leuven, Belgium). Library preparation was performed using the Illumina TruSeq Stranded mRNA Sample Preparation Kit, according to the manufacturer's protocol (Cat No. 20020594). RNA denaturation was performed at 65°C, followed by cool-down at 4°C. Subsequently, samples were indexed for multiplexing. Sequencing libraries were quantified using the Qubit fluorometer (Thermo Fisher Scientific, Massachusetts, USA). Library quality and size range were assessed using the Bioanalyzer (Agilent Technologies) with the DNA 1000 kit (Agilent Technologies, California, USA). Libraries were sequenced using the Illumina HiSeq4000 platform. 50 bp single-end reads and average of 20 million reads were obtained. Quality control of raw reads was performed with the FastQC v0.11.5. Filtering of adapters was performed using ea-utils v1.2.2.18. The splice-aware alignment was performed with TopHat v2.0.13 against the mouse genome mm10. The number of allowed mismatches was set to two and only unique mapping reads with alignment score of 10 or more were included. Resulting SAM and BAM alignment files were handled with Samtools v0.1.19.24. Quantification of reads per gene was performed with HT-Seq count v0.5.3p3. Count-based differential expression analysis was done using the R-based (The R Foundation for Statistical Computing, Vienna, Austria) Bioconductor package DESeq. Reported *P* values were adjusted for multiple testing with the Benjamini-Hochberg procedure, which controls false discovery rate (FDR).

### 2.10. ChIP Sequencing Analysis

Publicly available ChIP-seq data on the binding of Fra-1 in mouse embryonic fibroblasts was processed using the pipeline from the Kundaje lab (Version 0.3.3) [23]. Reads were aligned to reference genome (mm10) using Bowtie2 (v2.2.6) using the “--local” parameter. Single-end reads that aligned to the genome with mapping quality ≥30 were kept as usable reads (reads aligned to the mitochondrial genome were removed) using SAMtools (v1.2). PCR duplicates were removed using Picard's MarkDuplicates (Picard v1.126). Coverage was calculated using bamCoverage function with bin size = 1 bp and normalized using RPKM.

### 2.11. Statistical Analysis

Statistical analysis of RNA sequencing data statistical analysis was performed by bioconductor package DESeq (R-based). Reported *P* values were adjusted for multiple testing with the Benjamini-Hochberg procedure, which controls false discovery rate (FDR). In all the experiments, significant differences were determined by one-way analysis of variance (ANOVA), two-way ANOVA, adjusted for multiple comparison, and two-tailed unpaired *t*-test. Data are presented as the mean ± standard error of mean (SEM) or as fold change (FC). Data are visualized using GraphPad Prism 6 software (San Diego, CA, USA).

## 3. Results

### 3.1. AP-1 as a Predicted Regulator of Wound Healing-Associated Gene Expression in Fibroblasts

Together with immune cells, fibroblasts release growth factors as an immediate response to tissue damage, causing surrounding fibroblasts and keratinocytes to migrate towards the site of injury. To reestablish skin homeostasis, these cells cooperate to restore the wounded area with the formation of new tissue. This is achieved by cellular proliferation and migration as well as their capacity to remodel the extracellular matrix through continuous synthesis and breakdown of collagens [3, 6].

**Figure 1:**
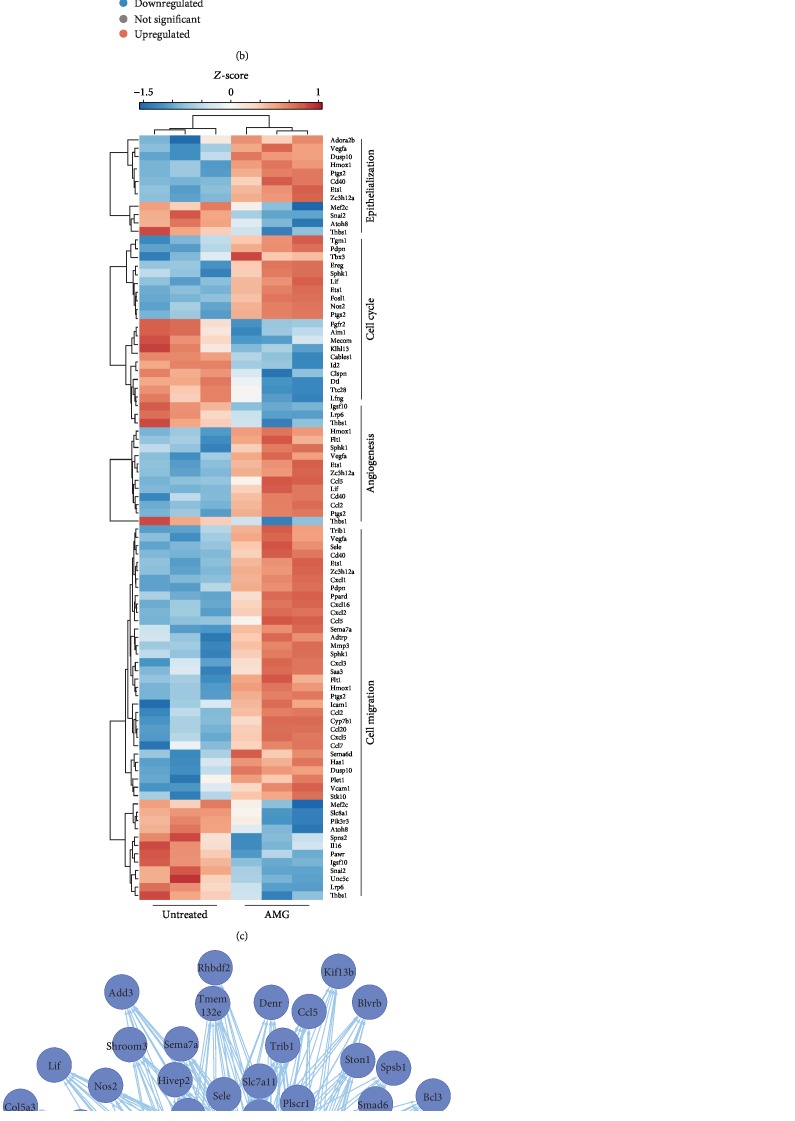
Predicted transcription factors and gene targets from a set of MG-induced DEGs. (a) Schematic representation of the transcriptional regulatory network analysis. Mouse primary fibroblasts isolated from tail's tip were used in the *in vitro* the scratch wound assay. MGs processed from a murine skin biopsy according to the Rigenera® protocol were processed to remove cellular remains and were subsequently applied to the *in vitro* scratch layers. Differentially expressed genes (DEGs) obtained via RNA-seq analysis (*N* = 3) were then used to identify the transcriptional regulatory networks using iRegulon. (b) Volcano plot showing up- (red) and downregulated (blue) genes upon 5 h of MG treatment. Black dots represent statistically insignificant genes. Gene values are reported as a log_2_ fold change (*P* value < 0.05). (c) Heatmap showing MG-dependent DEGs involved in essential wound healing process, such as epithelialization, cell cycle, angiogenesis, and cell migration. (d) Transcriptional regulatory network established around *Fosl1* using iRegulon, showing predicted transcription factors (light blue) to regulate their target genes (dark blue).

A recent study indicated that increased autologous micrograft- (AMG-) mediated fibroblast migration requires activation of the ERK signaling pathway that has been shown to be induced by the growth factors present in the soluble fraction of the micrograft [19]. To identify the transcriptional regulators mediating this behavior, we made use of the DEGs of wounded adult murine fibroblasts treated with the noncellular fraction of the micrograft treatment [19]. These genes were subsequently integrated in a gene regulatory network analysis using the iRegulon analysis software [24] (Figure 1(a)). The initial transcriptome analysis showed 123 upregulated DEGs (logFC > 1 , adjusted *P* value < 0.05), including genes involved in cell migration (i.e., *Cxcl2*, *Ccl20*, *Mmp3*), angiogenesis (i.e., *Ets1*, *Vegfa*, *Flt1*), cell cycle (i.e., *Nos2*, *Ptgs2*, *Lif*), and epithelialization (i.e., *Dusp10*, *Ptgs2*, *Hmox1*) (Figures 1(b) and 1(c), [Supplementary-material supplementary-material-1]).

The iRegulon analysis of AMG-dependent DEGs identified *Bcl3*, *Foxc2*, *Cebpg*, *Mms19*, *Foxo6*, *Crx*, *Fosl1*, *Otud4*, and *Plag1* as predicted transcription factors regulating DEG expression ([Supplementary-material supplementary-material-1]). Interestingly, among these, we identified AP-1 family member *Fosl1*, encoding the Fra-1 protein. AP-1 transcription factors have been reported to regulate the expression of genes encoding growth factors and MMPs [14]. Additionally, it has been shown that AP-1 activity is regulated by the ERK pathway [10, 25]. Prompted by this evidence, we decided to focus our attention on the role of the transcription factor AP-1 in regulating MG-mediated wound healing. Explicitly, our findings obtained from transcriptome analysis and gene regulatory network analysis suggested that AP-1 family member Fra-1 might retain a potential role in the regulation of the molecular mechanisms behind the beneficial effects of the soluble MG treatment (Figure 1(d)).

To validate the predicted binding of AP-1 as a transcriptional regulator to the promoter of wound healing-associated genes, we used publicly available ChIP-seq data to investigate the ability of the Fra-1 protein to bind the promoter region of several upregulated DEGs ([Supplementary-material supplementary-material-1]) [23]. Correspondingly, we confirmed binding of Fra-1 to the promoters of several genes, such as *Cxcl2*, *Ets1*, *Mmp13*, and *Fosl1* itself in mouse fibroblasts. Collectively, these findings provide strong evidence that the induced regenerative transcriptional profile in AMG-treated fibroblasts is likely regulated by the AP-1 family members.

### 3.2. Micrograft Treatment Induces AP-1 Activity via Increased *Fosl1* Expression and Fra-1 Phosphorylation

Studies have reported that the lack of AP-1 function in both fibroblasts and keratinocytes resulted in impaired cell migration and cell proliferation, and, in turn, led to delayed wound closure [14]. Therefore, we aimed to determine whether the noncellular MG treatment induces transcriptional activity of AP-1 through changes at transcriptional level or through posttranslational modifications in both cell types.

**Figure 2:**
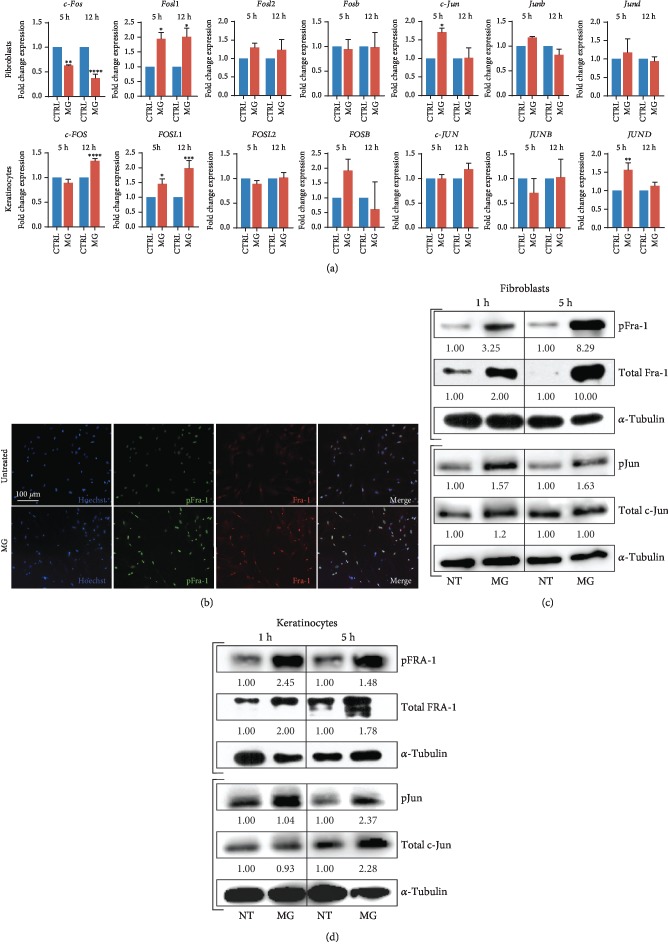
MG-dependent transcriptional and posttranscriptional regulation of AP-1 family members. (a) Transcript levels of AP-1 family members evaluated by RT-qPCR. Expression values are expressed as a fold change normalized to the expression of *Gapdh*, *β-actin*, and *Rpl13a* housekeeping genes. Differences are calculated with one-way ANOVA (*N* = 3) and indicated as ^∗^*P* < 0.05, ^∗∗^*P* < 0.01, ^∗∗∗^*P* < 0.001, and ^∗∗∗∗^*P* < 0.0001. (b) Representative immunofluorescence showing phosphorylation and total levels of Fra-1 protein in MG-treated and untreated primary adult fibroblasts. Nuclei were counterstained with Hoechst 33342 (*N* = 3). (c) Representative western blot membranes showing level of phosphorylated and total Fra-1 and c-Jun from wounded fibroblasts upon different time durations of MG treatment (1 h and 5 h). Untreated cells were used as a control. (d) Representative western blot membranes showing level of phosphorylated and total FRA-1 and c-JUN from MG-treated and MG-untreated human keratinocyte (HaCaT) cells exposed to 1 h and 5 h of MG treatment.

To determine the regulation of AP-1 activity upon soluble MG treatment, we assessed the transcript levels of its family members following 5 and 12 hours of MG treatment. In adult mouse fibroblasts as well as in the epithelial/keratinocyte (HaCaT) cell model, *Fosl1* and *FOSL1*, respectively, appeared as the sole AP-1 factor to be significantly upregulated upon treatment at the transcript level (Figure 2(a)). Although increased expression levels of Fos and Jun genes lead to higher activation of AP-1, posttranslational regulation of AP-1 family members also greatly contributes to its activity. As the DNA-binding properties of AP-1 member proteins and their transcriptional activity strongly depend on posttranslational phosphorylation, we evaluated the levels of phosphorylated Fra-1 (pFra-1) in mouse fibroblasts and FRA-1 (pFRA-1) in human keratinocytes after 1 and 5 h of MG treatment [11, 26]. As immunostaining and western blotting analysis revealed an increase in both total Fra-1 and phosphorylation of Fra-1 at serine 265 residues, this could indicate that newly formed Fra-1 proteins are immediately posttranslationally modified upon MG treatment (Figures 2(b), 2(c), and 2(d)).

Optimal transcriptional activity of Fra-1 is highly dependent of its binding to members of the Jun family. As the most potent transcriptional regulator of this family, *c-Jun* mRNA levels appear to be significantly increased upon 5 h of MG treatment in wounded murine adult fibroblasts (Figure 2(a)). Subsequently, we investigated whether c-Jun, as part of the AP-1 complex, was also posttranslationally regulated upon treatment with the micrograft solution. In both keratinocytes and fibroblasts, increased phosphorylation levels of c-Jun/c-JUN were observed after 1 h and 5 h of treatment (Figures 2(c) and 2(d)).

Taken together, we demonstrate that posttranslational activity of both Fra-1 and c-Jun in fibroblasts and keratinocytes is increased upon soluble MG treatment. This suggests that these AP-1 members are involved in the molecular mechanism of MG treatment and in the previously shown accelerated AMG-dependent wound healing [19].

### 3.3. Micrograft-Induced Cell Migration Is Reverted upon AP-1 Inhibition

**Figure 3:**
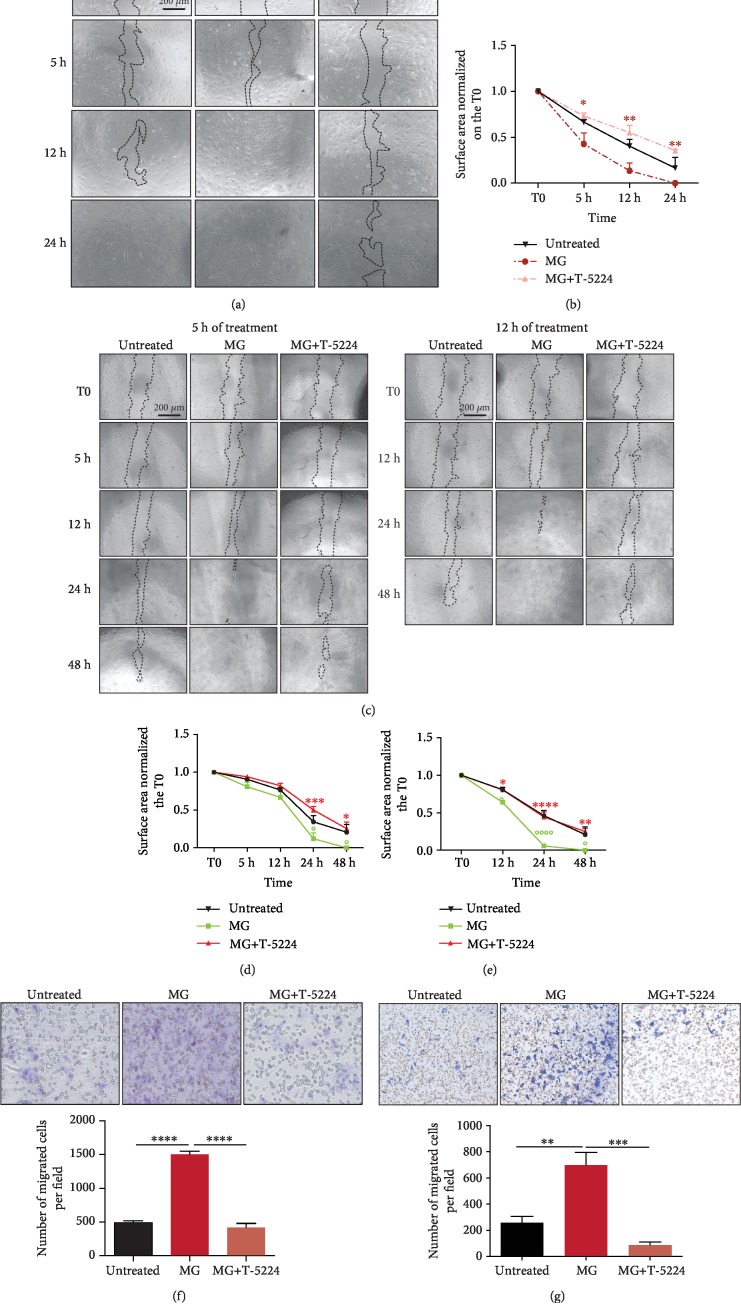
MG treatment accelerates scratch closure and cell migration through transcriptional activity of AP-1. (a) Representative images of wounded adult mouse fibroblasts exposed to 5 h of MG treatment in the presence or absence of the specific AP-1 inhibitor T-5224 (60 *μ*M). (b) Quantification of scratch closure from 3 independent experiments as in Figure 3(a). Data is presented as the mean ± SEM (*N* = 3). The surface area was normalized to the T0 image, and significant differences are calculated with two-way ANOVA, followed by the Holm-Sidak test and indicated as ^∗^*P* < 0.05; ^∗∗^*P* < 0.01; ^∗∗∗^*P* < 0.001. (c) Representative images of wounded human keratinocytes exposed to 5 h and 12 h of MG treatment in the presence or absence of AP-1 inhibitor T-5224 (60 *μ*M). (d, e) Quantification of scratch closure from 3 independent experiments as in Figure 3(c). Data is shown as the mean ± SEM (*N* = 3). The surface area was normalized to the T0 image and significant differences are calculated by two-way ANOVA followed by the Tukey test and indicated as ^∗^*P* < 0.05, ^∗∗^*P* < 0.01, ^∗∗∗^*P* < 0.005, and ^∗∗∗∗^*P* < 0.001. (f, g) Pictures showing transwell migration assays chamber membranes. Adult murine fibroblasts (left panel) and human (HaCaT) keratinocytes (right panel) were exposed to MG treatment in the presence or absence of the AP-1 inhibitor T-5224 (60 *μ*M). Data is presented as the mean ± SEM. *P* values were calculated using ordinary one-way ANOVA corrected by the Tukey test. ^∗^*P* < 0.05, ^∗∗^*P* < 0.01, ^∗∗∗^*P* < 0.001, ^∗∗∗∗^*P* < 0.0001.

Recently, it has been shown that the soluble fraction of autologous micrograft (AMG) treatment on wounded murine adult fibroblasts resulted in increased *in vitro* cell migration and MMP activity, leading to an improved *in vitro* scratch closure capacity and an improved *in vivo* wound healing [19]. As the predicted transcription factor AP-1 is known to regulate cell migration, we assessed its involvement in the accelerated MG-mediated scratch closure by evaluating the migratory capacity of MG-treated murine primary adult fibroblasts and human HaCaT cells using the *in vitro* cell scratch assay in the presence or absence of the specific c-Fos/AP-1 inhibitor T-5224 (Figures 3(a)–3(e)) [27]. AP-1 inhibition significantly delayed the *in vitro* scratch closure in MG-treated fibroblasts (Figures 3(a) and 3(b)). Similarly, MG-treated keratinocytes showed a significant delay in scratch closure following 5 h and 12 h of treatment in the presence of the AP-1 inhibitor, suggesting the essential role of AP-1 in regulating the migratory capacity of this cell type (Figures 3(c)–3(e)).

Interestingly, transwell migration assay of both MG-treated fibroblasts and keratinocytes showed significantly reduced cell migration upon AP-1 inhibition (Figures 3(f) and 3(g), respectively). This could suggest that AP-1 inhibition results in downregulation of essential chemokine receptors, leading to impaired cell migration. Collectively, we showed that inhibition of AP-1 directly resulted in impaired cell migration, completely reverting the effects of MG treatment on the migratory capacities in both fibroblasts and keratinocytes.

### 3.4. Micrograft-Accelerated Scratch Closure through AP-1 Activity Does Not Depend on Fibroblast Cell Proliferation

As the AP-1 family has been described to regulate both migration and proliferation [14], we assessed possible changes in cell cycle regulation in order to investigate the contribution of cell proliferation to the *in vitro* scratch closure.

**Figure 4:**
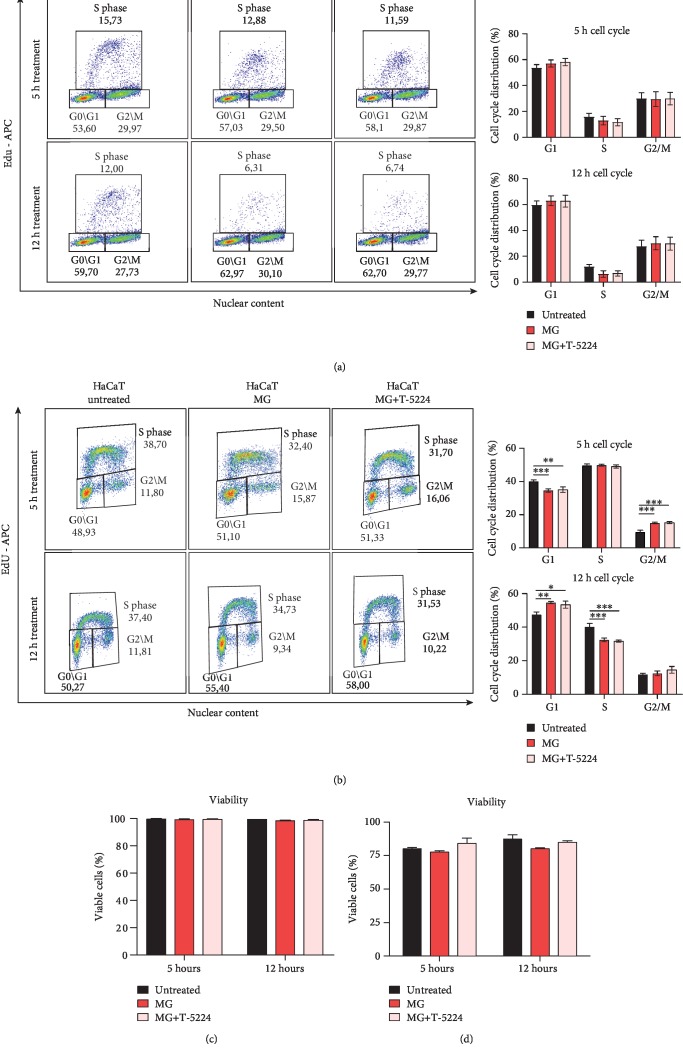
AP-1 in MG-treated cells does not affect fibroblast cell proliferation and viability. (a, b) Cell cycle changes were analyzed using the common 5-ethynyl-2′-deoxyuridine (EdU) assay (associated with DAPI staining to evaluate the nuclear content) on both MG-treated fibroblasts and human keratinocytes exposed to 5 and 12 h of MG treatment in the presence or absence of the AP-1 inhibitor T-5224. Quantification average of *N* = 3 is shown. Data is presented as the mean ± SEM. Two-way ANOVA was used to perform statistical analysis. ^∗^*P* < 0.05; ^∗∗^*P* < 0.01; ^∗∗∗^*P* < 0.001. (c, d) MG-induced effects on cell viability were evaluated via FACS analysis using the Fixable Viability Stain 660. Percentage (%) of viable cells after exposure to 5 and 12 h of MG treatment is shown. No significant differences were found among the different conditions.

To determine the role of AP-1 in cell proliferation under cell-free MG-treated conditions, we performed a cell cycle assessment on soluble MG-treated proliferating fibroblasts and keratinocytes using the 5-ethynyl-2′-deoxyuridine (EdU) assay in the presence of the AP-1 inhibitor T-5224. Interestingly, no changes in cell cycle phases were observed in MG-treated fibroblasts and the overall proliferation rate remained unchanged (Figure 4(a)). In contrast, MG-treated keratinocytes showed a decreased number of cells in the S-phase, indicating reduced cell proliferation of keratinocytes upon MG treatment (Figure 4(b)). Furthermore, we showed that MG treatment upon AP-1 inhibition does not alter cell viability in both cell types (Figures 4(c) and 4(d)). Therefore, we exclude the involvement of AP-1 in promoting cell proliferation upon soluble MG treatment *in vitro*.

### 3.5. Micrograft Treatment Regulates the Matrix Remodeling Machinery in an AP-1-Dependent Manner

**Figure 5:**
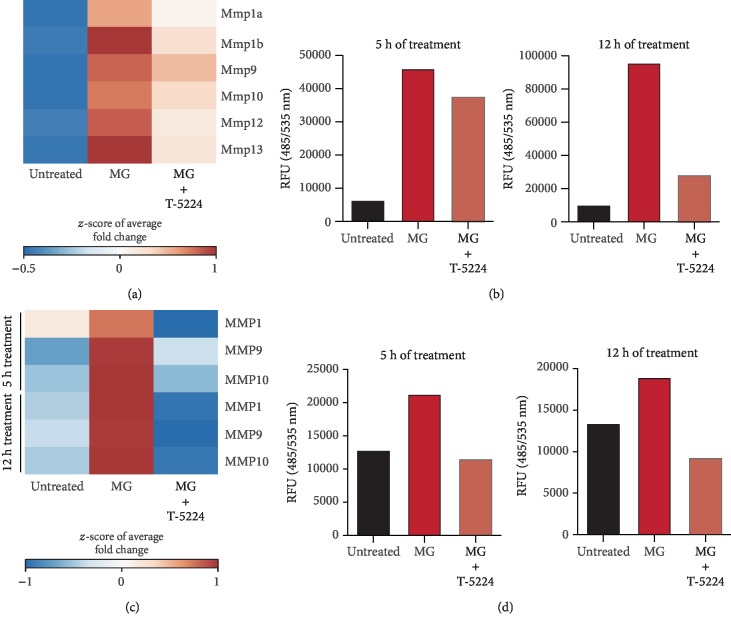
Transcriptional and posttranscriptional regulation of the matrix remodeling machinery via MG-dependent AP-1 activity. (a) Matrix metalloproteinase (MMP) gene expression levels were evaluated by RT-qPCR in 5 h MG-treated adult fibroblasts in the presence or absence of T-5224 (60 *μ*M). Expression values are expressed as *z*-score of the average fold change (FC) normalized to the expression of *GAPDH*, *β-actin*, and *Rpl13a* genes (*N* = 3). (b) Enzymatic activity of MMPs in adult fibroblasts exposed to 5 and 12 h of MG treatment was evaluated via FRET (fluorescence resonance energy transfer). Signal intensities were normalized to the substrate control. Data is reported as relative fluorescence units (RFU). (c) MMP gene expression levels were evaluated by RT-qPCR in 5 h and 12 h MG-treated human keratinocytes in the presence or absence of T-5224 (60 *μ*M). Expression values are expressed as *z*-score of the average FC normalized on the expression of *GAPDH*, *β-actin*, and *RPL13A* genes (*N* = 3). (d) Enzymatic activity of matrix metalloproteinases of conditioned media derived from human keratinocytes exposed to 5 and 12 h of MG treatment was evaluated via FRET (frequency resonance energy transfer). Signal intensity signals were normalized to the substrate control. Data is reported as relative fluorescence units (RFU).

During the healing process, cell migration and active extracellular matrix (ECM) remodeling represent crucial events for successful wound healing. Therefore, we aimed to assess the impact of AP-1 activity on the enzymatic function of MMPs. Recently, it has been shown that essential wound healing-associated genes, such as cytokines and MMPs, were strongly upregulated upon AMG treatment [19]. Upon transcriptional analysis of this dataset, these in turn appeared to be targets of the AP-1 (Figures 1(b) and 1(d) and [Supplementary-material supplementary-material-1]). Additionally, it has previously been shown that upon AMG treatment, MMP expression levels and their enzymatic activity were significantly increased [19]. As AP-1 has been reported to regulate the expression levels of several genes, including those encoding MMPs [14], we determined whether the soluble MG-induced activity of MMPs was AP-1 dependent. Cell-free MG-treated fibroblasts and keratinocytes were cocultured in the presence of the AP-1 inhibitor T-5224. Interestingly, we observed that AP-1 inhibition in MG-treated fibroblasts and keratinocytes expressed significantly lower transcript levels of several MMP members after 5 h and 12 h of treatment (Figures 5(a) and 5(c)). Subsequently, we exposed fibroblasts and keratinocytes in the *in vitro* scratch assay to the MG solution treatment with and without AP-1 inhibition. The conditioned medium collected from these conditions was then used to perform a fluorescence-based assay to determine the enzymatic activity of the MMPs. Remarkably, enzymatic activity was greatly hindered upon AP-1 inhibition in fibroblasts and keratinocytes (Figures 5(b) and 5(d)). These findings indicate that AP-1 might play a central role in MG-induced cell migration, and more importantly, this connection might explain the improved wound healing upon cell-free MG treatment.

### 3.6. AP-1 Transcriptional Activity Is Responsible for ERK-Mediated Wound Healing upon Micrograft Treatment

Wound healing processes enhanced by the soluble MG have been shown to be induced in an ERK-dependent manner [19]. To further unravel the downstream mechanism of this treatment at the transcriptional level, we showed the importance of the transcription factor AP-1 in regulating cell migration and MMP activity upon MG treatment. As it has been reported that AP-1 is regulated by ERK, we assessed whether this regulation takes place in both MG-treated fibroblasts and keratinocytes [11, 12]. Therefore, we determined posttranslational modification of important AP-1 family members, including phosphorylation of the mouse and human homologs of Fra-1 and c-Jun in the presence of the MEK inhibitor PD0325901.

**Figure 6:**
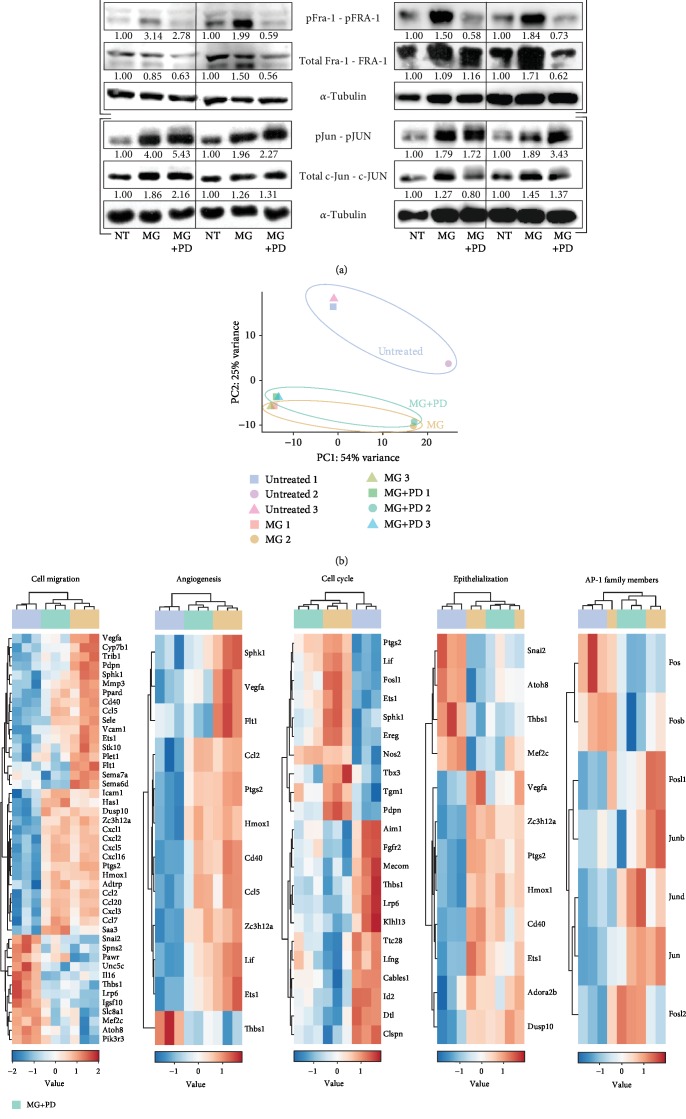
ERK signaling pathway activation regulates wound healing events through AP-1 transcription factor activity. (a) Representative western blot showing protein levels of phosphorylated and total ERK, Fra-1, and c-Jun from wounded murine adult fibroblasts and unwounded human keratinocytes (HaCaT cells) exposed to 1 h and 5 h of MG treatment in the presence or absence of the MEK inhibitor PD0325901 (1 *μ*M). (b) Principal component analysis (PCA) plot obtained from RNA-seq data indicates clustering between untreated and MG-treated samples with or without MEK inhibition. (c) Heatmaps showing gene expression levels of DEGs discovered through RNA-seq analysis are shown to be involved in essential wound healing related events, including cell migration, angiogenesis, cell cycle, and epithelialization. Gene expression levels of AP-1 family members are presented as heatmap in all the conditions under study.

Interestingly, upon ERK inhibition in MG-treated cells, reduced levels of pFra-1/pFRA-1 were observed while pJun/pJUN did not decrease (Figure 6(a)). This could suggest that heterodimers involving Fra-1 regulate the ERK-dependent effects of the MG treatment. To validate this, we performed RNA sequencing on MG-treated wounded fibroblasts in the presence or absence of the MEK inhibitor PD0325901 (Figure 6(b)). Using DEG-derived GO terms, expression values of genes involved in cell migration, angiogenesis, cell cycle, and epithelialization showed clear transcriptional changes upon MEK inhibition (Figure 6(c)). As these genes are known to be differentially expressed upon micrograft treatment [19], we assessed whether these genes were affected by MEK inhibition. Although a few crucial genes of angiogenesis (e.g., *Vegfa* and *Flt1*) were altered by this inhibition, a multitude of genes involved in cell migration were significantly affected upon treatment with PD0325901, confirming that the soluble MG treatment mainly increased cell migration. Additionally, high levels of *Fosl1* and *Junb* were downregulated after MEK inhibition, possibly linking this reduction to the changes in cell migration.

## 4. Discussion

As skin injuries and impaired tissue regeneration have drastic financial and medical consequences for patients, micrografts (MG) provide an affordable treatment to aid the endogenous wound healing. Although multiple MG systems have been shown to be successful in clinical trials, the molecular mechanisms behind these techniques remain unclear [16]. Previously, it has been demonstrated that cell-free autologous MG (AMG) treatment improves ERK-dependent cell migration and matrix remodeling, resulting in accelerated wound closure *in vitro* and *in vivo* [19]. However, the downstream mechanisms of the transcriptional regulation behind the soluble MG-induced cellular changes have not yet been explored.

In the present work, we identified AP-1 among other transcription factors responsible for establishing a regenerative transcriptional profile in both fibroblasts (Figure 1) and keratinocytes. Although inhibition of AP-1 activity leads to a drastic reduction in cell migration and loss of a transcriptional program favoring matrix remodeling, we do not exclude indirect effects triggered by AP-1 nor the contribution of other essential transcription factors to the improved soluble MG-induced wound healing. As another predicted regulator of the identified DEGs, B cell lymphoma 3 (Bcl3) has been shown to promote wound healing as its knockdown resulted in significantly reduced cell proliferation and migration in the human osteosarcoma U2OS line [28]. Additionally, keratinocyte migration has been demonstrated to be drastically impaired upon *CAAT enhancer-binding protein gamma* (*C/EBPγ*) silencing [29]. Aside from cell migration, dysregulated genes involved in other wound healing processes might be regulated by other predicted TFs. For example, *Forkhead box protein C2* (*Foxc2*) is a known regulator of angiogenesis in both wound healing and oral squamous cell carcinoma [30, 31]. However, as AP-1 is known to regulate both MMP activity and cell migration, and is additionally regulated by ERK signaling, we aimed our attention at the AP-1 complex member *Fosl1*.

Upon cell-free MG treatment, both *Fosl1*/*FOSL1* transcript levels and phosphorylated Fra-1 and FRA-1 contribute to an elevated AP-1 activity in both fibroblasts and keratinocytes (Figures 2(a), 2(c), and 2(d)). The activator protein-1 (AP-1) complex is composed of homo- and heterodimers of leucine zipper proteins where a Fos family member binds to a protein of the Jun family [32]. While the Jun family is able to form homodimers, Fos proteins are restricted to dimerization with a Jun member [33]. Upon dimerization, AP-1 binds to 12-O-tetradecanoate-13-acetate (TPA) response elements (TRE) within the genome, although its transcriptional behavior strongly depends on the dimer composition and its phosphorylation [13]. As Fra-1 is likely to form the AP-1 complex by dimerization with the most potent Jun protein c-Jun, c-Jun phosphorylation (pJun) found to be increased upon soluble MG treatment. While it has been reported that *c-Jun* expression is minimally affected after *in vitro* wounding, elevated mRNA levels of Fos genes are found shortly after injury [34]. Among these Fos members, *c-Fos* and *Fosl1* have been reported to be briefly expressed as an immediate response to extracellular stimuli, favoring the wound recovery by initiating the expression of healing-associated genes, such as growth factors, MMPs, and integrins [14]. Accordingly, upon specific AP-1 inhibition, the soluble MG-induced scratch closure is completely reverted, and the cellular migratory capacity showed to be highly impaired (Figure 3). Of note, a slight MG-dependent reduction in proliferation was displayed in treated keratinocytes. However, as MG treatment as well as AP-1 induction did not increase cell proliferation, we affirm that changes in cell cycle did not contribute to accelerated *in vitro* wound closure in an AP-1-dependent way (Figure 4). In addition, the enzymatic activity of matrix remodeling enzymes (MMPs) was severely compromised in the presence of AP-1 inhibitor T-5224 (Figure 5). Interestingly, decreased Fra-1 expression has been reported to result in decreased MMP expression and cell migration [35, 36]. Moreover, overexpression of Fra-1 significantly improves cell motility and invasiveness in cancer [37, 38]. Taken together, these findings support the role of Fra-1/AP-1 in regulating cell migration in the context of wound healing as matrix remodeling and integrin expression facilitates cellular movement through the matrix.

Transcriptional regulation of Fos proteins occurs mainly through MAPK activity as a response to mitogenic cues. As a response to extracellular signals, ternary complex factor (TCF) phosphorylated by ERK enables its binding together with SRE-binding factor (SRF) to serum response elements (SRE) in the promoter region of *Fos* genes. Furthermore, it has been reported that AP-1 is able to autoregulate itself by binding to promoter regions of *Fos* genes and *c-Jun* [39–41]. In addition to transcriptional regulation, AP-1 members are posttranscriptionally phosphorylated by MAPK components, leading to reduced ubiquitination and increased transcriptional activity [42, 43]. Studies have shown that JNK-mediated phosphorylation of the serine 63 and 73 residues of c-Jun increases both stability and activity [42, 44]. Similarly, Fra-1 stability depends on phosphorylation on serine 252 and 265 by ERK1/2 signaling [45]. In addition, phosphorylation by ERK1/2 results in higher activity of Fos proteins [46, 47]. In this work, we confirm that MG-induced phosphorylation of Fra-1 is strongly dependent on ERK1/2 activity in both fibroblasts and keratinocytes (Figure 6(a)). Additionally, we report that both phosphorylated and total Fra-1 levels decrease in the presence of the ERK inhibitor, confirming the ERK-dependent posttranscriptional regulation of this *Fosl1-*encoded protein. Noteworthy, cell-free MG-induced phosphorylation levels of c-Jun appear to be independent of ERK, suggesting its regulation through other mechanisms. As mentioned earlier, the JNK branch of the MAPK cascade could be responsible for this increase, as JNK contributes to AP-1 expression upon exposure to mechanical stress, in this case the *in vitro* wounding [48]. Finally, RNA-seq data indicate that ERK inhibition greatly impairs the migratory transcriptional profiles of MG-treated adult fibroblasts. As most of these DEGs are predicted to be regulated by AP-1 (Figure 1), this could suggest that soluble MG-enhanced cell migration arises as a result of the transcriptional activity of AP-1.

**Figure 7:**
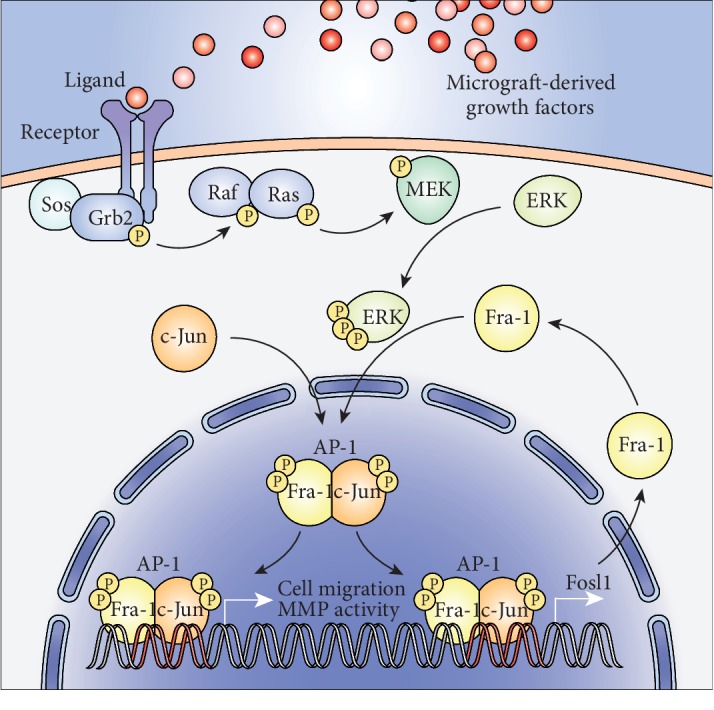
Schematic representation of the ERK-mediated mechanisms of MG treatment. Upon MG treatment, healing-associated growth factors present in the soluble fraction of the micrograft activate the MAPK signaling pathway, resulting in the phosphorylation of ERK. c-Jun and Fra-1 undergo dimerization to form the transcription factor AP-1. Once phosphorylated by ERK, AP-1 stabilizes in the nucleus to bind promoter regions of *Fosl1*, establishing a feedback loop. Additionally, AP-1 also binds the promoter regions of wound healing-associated genes, such as those involved in the regulation of cell migration.

Collectively, we propose a mode of action behind the accelerated wound healing using the cell-free MG treatment (Figure 7). Our findings indicate that soluble MG-induced ERK signaling has a double mechanism to regulate matrix remodeling and cell migration through transcriptional activity of AP-1. As ERK signaling is activated upon MG solution treatment, this cascade leads to the increased gene expression of *Fosl1*, resulting in a higher amount of the transcription factor Fra-1/AP-1. This in turn becomes posttranscriptionally phosphorylated by the active ERK signaling. Phosphorylated AP-1 subsequently triggers a migratory phenotype in keratinocytes and fibroblasts through the activation of MMP activity and a transcription program favoring chemotaxis and cell motility. Although other signaling pathways and transcription factors are likely to contribute to this phenotype, we show that inhibition of both ERK and AP-1 is sufficient to completely revert *in vitro* wound closure by the soluble MG treatment. Altogether, our results highlight the crucial role of ERK-dependent transcriptional activity of AP-1 in coordinating the beneficial effects of the MG treatment, opening new opportunities for future wound healing studies.

## Data Availability

The data presented in this paper has been deposited in GEO Datasets under accession no. GSE134113.

## Supplementary Materials

Supplementary 1 Table S1: primer sequences used to determine mRNA levels in mouse fibroblasts and human keratinocytes. Sequences are represented in the 5′-3′ direction.

Supplementary 2 Table S2: list of differentially expressed genes (unregulated, downregulated, and nondifferentially expressed) upon 5 h of micrograft treatment (as represented in Figure 1).

Supplementary 3 Figure S1: (A) Predicted transcription factors identified by iRegulon as possible regulators of the DEGs discovered in MG-treated conditions. Transcription factor prediction is presented as network enrichment score (NES). (B) ChIP-seq showing the binding of Fra-1/AP-1 in the promoter regions of several upregulated DEGs involved in wound healing processes, predicted to be regulated by AP-1 in the iRegulon analysis.
