# Supplementary material for: Activator Protein-1 Transcriptional Activity Drives Soluble Micrograft-Mediated Cell Migration and Promotes the Matrix Remodeling Machinery
Source: Stem Cells Int. 2019 Dec 31;2019:6461580. doi: 10.1155/2019/6461580 (PMC7012246; doi:10.1155/2019/6461580)
Supplement: Supplementary 1 — Table S1: primer sequences used to determine mRNA levels in mouse fibroblasts and human keratinocytes. Sequences are represented in the 5′-3′ direction. [file 6461580.f1.pdf]

MOUSE		
**GENE**	**FORWARD SEQUENCE**	**REVERSE SEQUENCE**
Fos	GGGAATGGTGAAGACCGTGTCA	GCAGCCATCTTATTCCGTTCCC
Fosb	ACCTGTCTTCGGTGGACTCCTT	TGGCTGGTTGTGATTGCGGTGA
Fosl1	CCGAAGAAAGGAGCTGACAGAC	CTCAAGGCGTTCCTTCTGCTTC
Fosl2	AGGAGGAGAAGCGTCGAATCCG	CCAGACTTCTCCTCTTCCAGCT
Jun	CAGTCCAGCAATGGGCACATCA	GGAAGCGTGTTCTGGCTATGCA
Junb	GACCTGCACAAGATGAACCACG	ACTGCTGAGGTTGGTGTAGACG
Jund	ACCTGCACAAGCAAAGCCAGCT	CGAAACTGCTCAGGTTGGCGTA
Mmp1a	AGGAAGGCGATATTGTGCTCTCC	TGGCTGGAAAGTGTGAGCAAGC
Mmp1b	GCAGTTGTGGAAGATGCCATCG	CCATCAAATGTGTAGAAGTCACC
Mmp9	GGACCCGAAGCGGACATTG	CGTCGTCGAAATGGGCATCT
Mmp10	TGCTGCCTATGAGGCTCACAAC	GGAGGAAAACCGAGAGTGTGGA
Mmp12	CACACTTCCCAGGAATCAAGCC	TTTGGTGACACGACGGAACAGG
Mmp13	GATGACCTGTCTGAGGAAGACC	GCATTTCTCGGAGCCTGTCAAC
**HUMAN**		
GENE	**FORWARD SEQUENCE**	**REVERSE SEQUENCE**
FOS	GCCTCTCTTACTACCACTCACC	AGATGGCAGTGACCGTGGGAAT
FOSB	TCTGTCTTCGGTGGACTCCTTC	GTTGCACAAGCCACTGGAGGTC
FOSL1	GGAGGAAGGAACTGACCGACTT	CTCTAGGCGCTCCTTCTGCTTC
FOSL2	AAGAGGAGGAGAAGCGTCGCAT	GCTCAGCAATCTCCTTCTGCAG
JUN	CCTTGAAAGCTCAGAACTCGGAG	TGCTGCGTTAGCATGAGTTGGC
JUNB	CGATCTGCACAAGATGAACCACG	CTGCTGAGGTTGGTGTAAACGG
JUND	ATCGACATGGACACGCAGGAGC	CTCCGTGTTCTGACTCTTGAGG
MMP1	ATGAAGCAGCCCAGATGTGGAG	TGGTCCACATCTGCTCTTGGCA
MMP9	GCCACTACTGTGCCTTTGAGTC	CCCTCAGAGAATCGCCAGTACT
MMP10	TCCAGGCTGTATGAAGGAGAGG	GGTAGGCATGAGCCAAACTGTG
