# Supplementary figures and images for: Activator Protein-1 Transcriptional Activity Drives Soluble Micrograft-Mediated Cell Migration and Promotes the Matrix Remodeling Machinery
Source: Stem Cells Int. 2019 Dec 31;2019:6461580. doi: 10.1155/2019/6461580 (PMC7012246; doi:10.1155/2019/6461580)

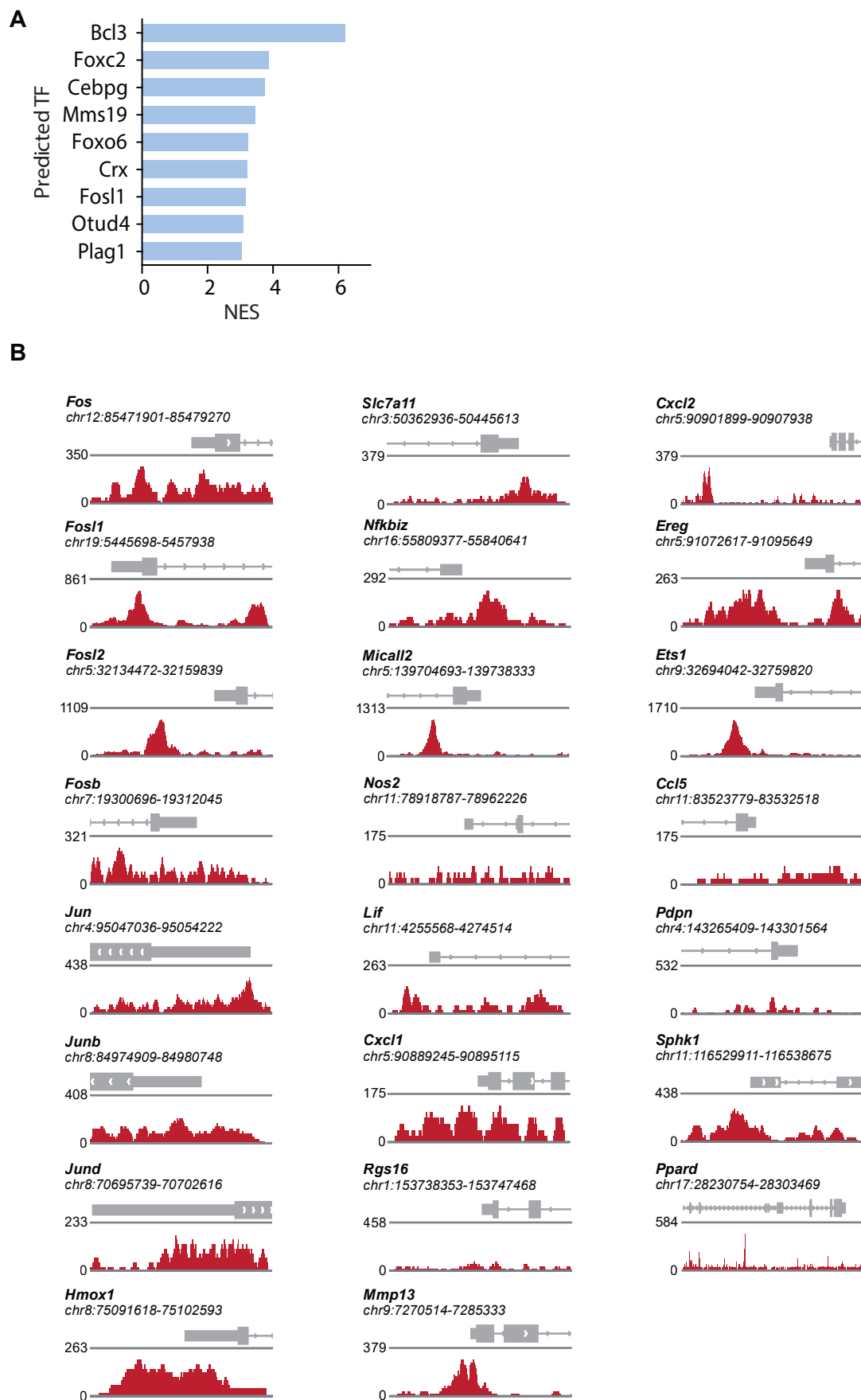

Figure S1 - Balli, Chui et al. 2019

Supplement: Supplementary 3 — Figure S1: (A) Predicted transcription factors identified by iRegulon as possible regulators of the DEGs discovered in MG-treated conditions. Transcription factor prediction is presented as network enrichment score (NES). (B) ChIP-seq showing the binding of Fra-1/AP-1 in the promoter regions of several upregulated DEGs involved in wound healing processes, predicted to be regulated by AP-1 in the iRegulon analysis. [file 6461580.f3.pdf]
